# Diamondoids are not forever: microbial biotransformation of diamondoid carboxylic acids

**DOI:** 10.1111/1751-7915.13500

**Published:** 2019-11-12

**Authors:** Benjamin D. Folwell, Terry J. McGenity, Corinne Whitby

**Affiliations:** ^1^ School of Life Sciences University of Essex Wivenhoe Park Colchester CO4 3SQ UK; ^2^ Present address: Informa 110 Southwark St London SE1 0TA UK

## Abstract

Oil sands process‐affected waters (OSPW) contain persistent, toxic naphthenic acids (NAs), including the abundant yet little‐studied diamondoid carboxylic acids. Therefore, we investigated the aerobic microbial biotransformation of two of the most abundant, chronically toxic and environmentally relevant diamondoid carboxylic acids: adamantane‐1‐carboxylic acid (A1CA) and 3‐ethyl adamantane carboxylic acid (3EA). We inoculated into minimal salts media with diamondoid carboxylic acids as sole carbon and energy source two samples: (i) a surface water sample (designated TPW) collected from a test pit from the Mildred Lake Settling Basin and (ii) a water sample (designated 2 m) collected at a water depth of 2 m from a tailings pond. By day 33, in TPW enrichments, 71% of A1CA and 50% of 3EA was transformed, with 50% reduction in EC_20_ toxicity. Similar results were found for 2 m enrichments. Biotransformation of A1CA and 3EA resulted in the production of two metabolites, tentatively identified as 2‐hydroxyadamantane‐1‐carboxylic acid and 3‐ethyladamantane‐2‐ol respectively. Accumulation of both metabolites was less than the loss of the parent compound, indicating that they would have continued to be transformed beyond 33 days and not accumulate as dead‐end metabolites. There were shifts in bacterial community composition during biotransformation, with *Pseudomonas* species*,* especially *P. stutzeri,* dominating enrichments irrespective of the diamondoid carboxylic acid. In conclusion, we demonstrated the microbial biotransformation of two diamondoid carboxylic acids, which has potential application for their removal and detoxification from vast OSPW that are a major environmental threat.

## Graphical abstract

Microbes from OSPW aerobically transformed two model diamondoid carboxylic acids. 2‐hydroxy adamantane‐1‐carboxylic acid, and 3‐ethyl adamantane‐2‐ol were produced. Toxicity reduced during microbial biotransformation of diamondoid carboxylic acids. *Pseudomonas potentially* involved in diamondoid carboxylic acid transformation.

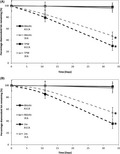

## Introduction

The bacterial oxidation of petroleum hydrocarbons over geological time has resulted in the formation of vast oil sands deposits, such as those in northern Alberta, Canada, which contain approximately 270 × 10^9^ m^3^ (1.6 × 10^12^ billion barrels) of bitumen (Chalaturnyk *et al.*, 2002). Bitumen extraction from surface‐mined oil sands produces large quantities of oil sands process‐affected water (OSPW), which are stored in tailings ponds. One of the major challenges associated with OSPW is the presence of organic acids, collectively known as naphthenic acids (NAs) (Whitby, 2010; Skeels and Whitby, 2018). Naphthenic acids are the main toxic components of OSPW and demonstrate both acute and chronic toxicity to a wide variety of species including plants (Kamaluddin and Zwaizek, 2002), fish (Nero *et al.*, 2006), zooplankton (Dokholyan and Magomedov, 1984) and bacteria (*Aliivibrio fischeri*) (Frank *et al.*, 2008). Higher molecular weight NAs often have greater acute toxicity (Holowenko *et al.*, 2002). NAs found in OSPW were poorly defined, due to their structural complexity (Headley *et al.*, 2016). NAs were classically described to have the general chemical formula C*_n_*H_2_ *_n_*_+_ *_Z_*O_2,_ where *n* is the number of carbon atoms and *Z* is either zero or a negative integer representing the number of hydrogen atoms lost due to ring formation (Whitby, 2010). However, NA structures that contain more than two oxygen atoms and/or other heteroatoms, such as nitrogen and sulphur, have also been found to be major components of NA mixtures in wastewaters (Wang *et al.*, 2013; West *et al.*, 2013). In addition, an extensive series of previously overlooked tri‐, tetra‐ and pentacyclic naphthenic acids were identified as major components of oil sands and OSPW (Rowland *et al.*, 2011a, 2012). These included the tricyclic diamondoid carboxylic acids, adamantane‐1‐carboxylic acid (A1CA) and 3‐ethyl adamantane carboxylic acid (3EA), which were investigated in our study. Tricyclic (and bicyclic) diamondoid carboxylic acids are major components of OSPW, and unrefined oil sands bitumen are reported to contain 90% tricyclic acids (Rowland *et al*, 2011b; Wilde *et al*, 2015). Both A1CA and 3EA exhibit chronic toxicity and specifically were found to disrupt human liver enzyme activity (Scarlett *et al.*, 2012). Currently, there are no cost‐effective and environmentally non‐invasive routes for the removal of these diamondoid (tricyclic) NAs from the environment. Diamondoid carboxylic acids are therefore of great environmental and human‐health significance and were thus selected as model compounds in our biotransformation studies.

Although many microorganisms have been found to biodegrade single‐ringed NAs (reviewed in Whitby, 2010; Skeels and Whitby, 2018), the higher molecular weight compounds, including the highly branched and multi‐ringed diamondoid NAs are more difficult to degrade (Scott *et al.*, 2005; Johnson *et al.*, 2011; Demeter *et al.*, 2015; Ahad *et al.*, 2018). However, some isolates have been found to have a limited capacity for growth on diamondoid acids, and bacteria belonging to the genera *Ochrobactrum* and *Bacillus* have been shown to metabolize surrogates of tricyclic NAs (Yue *et al.*, 2015). Furthermore, algae of the order *Chlorellales* and genus *Acutodesmus* dominated enrichments amended with A1CA, suggesting that microalgal‐bacterial communities have the potential to degrade NAs in tailings ponds (Paulssen and Gieg, 2019). It is currently unknown whether other microorganisms can degrade diamondoid carboxylic acids found in OSPW. Here, A1CA and 3EA were used as model diamondoid carboxylic acids to determine whether microorganisms derived from OSPW were capable of their biotransformation and to identify any metabolites produced, and the microorganisms potentially responsible.

## Results and discussion

### Biotransformation of diamondoid carboxylic acids by OSPW communities

**Figure 1:**
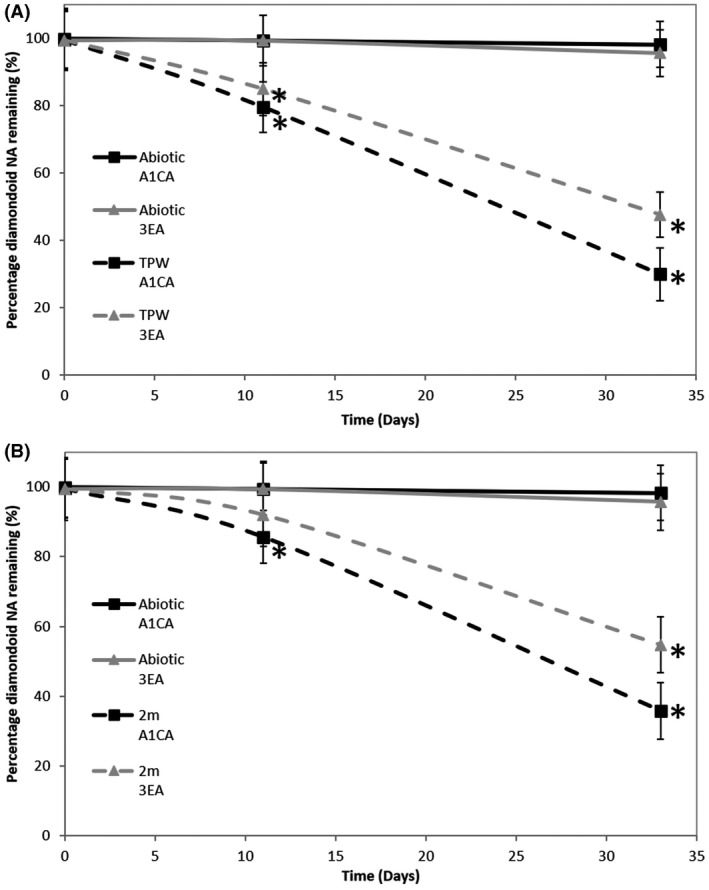
Biotransformation of diamondoid NAs by the microbial communities derived from the TPW (A) and 2 m (B) OSPW samples. Error bars represent the standard error of the mean (*n* = 3). *Significant removal compared to abiotic control (Mann–Whitney *U* test; *p < *0.05).

We investigated the aerobic biotransformation of two model diamondoid carboxylic acids, A1CA and 3EA, by microorganisms derived from OSPW, with a concomitant toxicity reduction. Our results showed that compared to the abiotic controls, the microbial community derived from an OSPW sample (denoted TPW), transformed ~ 20% of A1CA by day 11 and 71% by day 33; whilst the microbial community derived from another OSPW sample (denoted 2 m) transformed 15% of A1CA by day 11 and 64% by day 33 (Fig. 1B). Analysis of enrichments amended with 3EA showed that the microbial community derived from TPW had transformed 15% of 3EA by day 11 and ~ 52% by day 33 (Fig. 1A). In contrast to the TPW community, the microbial community derived from the 2 m sample showed no significant removal of the 3EA by day 11 but had removed ~ 46% by day 33 compared to the abiotic control (Fig. 1B). At day 33, 20% more A1CA had been transformed compared to 3EA by both the TPW and 2 m communities (Fig. 1).

Microbial biodegradation of aliphatic and aromatic carboxylic acids has been demonstrated previously (Smith *et al.*, 2008; Johnson *et al.*, 2011, 2012). Bacteria belonging to the genera *Ochrobactrum* and *Bacillus* have been shown to metabolize A1CA, albeit at a modest level (Yue *et al.*, 2015). Recently, Paulssen and Gieg (2019) showed that mixed cultures of algae‐bacteria removed up to 80% A1CA between 30 and 90 days, at comparable rates to the present study. In contrast, other studies investigating NA biodegradation by biofilms found no evidence of A1CA biodegradation (Demeter *et al.*, 2015). Furthermore, under low oxygen (Ahad *et al.*, 2018) and anaerobic (Folwell *et al.*, 2015) conditions, A1CA was found to persist despite the incubation conditions being amenable for the biodegradation of other compounds such as cyclohexanecarboxylic acid and 1,2‐cyclohexanedicarboxylic acid (Ahad *et al.*, 2018) or 2‐methylnaphthalene (Folwell *et al.*, 2015). In addition, the biotransformation of A1CA in our study was faster (although non‐significantly so) than the more‐branched 3EA. These findings are consistent with previous studies showing that NA biotransformation rates are influenced by molecular weight, chemical structure and specifically the degree of alkyl‐side chain branching (Herman *et al.*, 1994; Scott *et al.*, 2005; Smith *et al.*, 2008; Johnson *et al.*, 2011).

**Figure 2:**
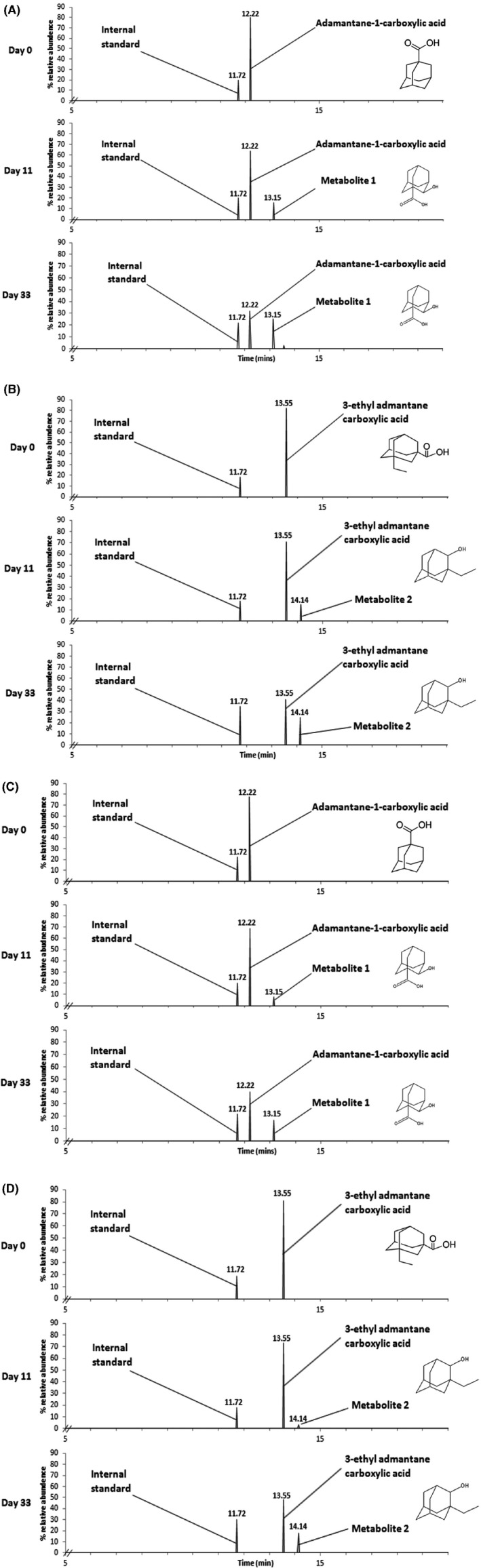
Gas chromatograms showing the transformation of the diamondoid NAs over a 33‐day incubation period by the microbial community derived from TPW or 2 m. TPW enrichments grown on A1CA (A) and 3EA (B) and 2 m enrichments grown on A1CA (C) and 3EA (D).

**Figure 3:**
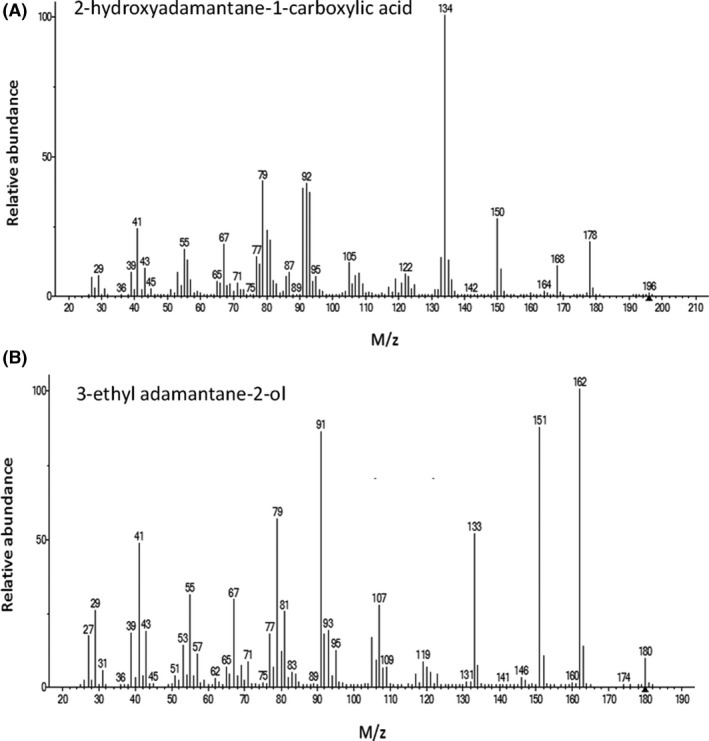
Mass spectra of metabolites produced during transformation of diamondoid carboxylic acids. Mass spectrum of metabolite 1 (2‐hydroxy‐adamantane‐1‐carboxylic acid) produced during the transformation of A1CA (A) and mass spectrum of metabolite 2 (3‐ethyl adamantane‐2‐ol) produced during the transformation of 3EA (B).

While studies have reported the biodegradation of A1CA (Paulssen and Gieg, 2019), the presence of metabolites has not been shown previously. In our study, the aerobic microbial biotransformation of A1CA and 3EA by the TPW and 2 m communities resulted in the production of two metabolites by day 11. Metabolite 1 (2‐hydroxyadamantane‐1‐carboxylic acid) derived from the biotransformation of A1CA had a retention time of 13.15 min, and metabolite 2 (3‐ethyladamantane‐2‐ol) produced during the biotransformation of 3EA had a retention time of 14.14 min, and both were detected irrespective of the inoculum (Fig. 2). No metabolites were detected in either the abiotic or killed controls. Mass spectra of each metabolite are shown in Fig. 3 and were present in similar abundance (relative to the internal standard) at day 11 and day 33, while the parent carboxylic acid decreased in abundance during the same period (Fig. 2). Under aerobic conditions, Selifonov (1992) showed that after growth on the bicyclic terpene camphor, washed cells of *P. putida* PpG1 were able to transform adamantanone into a number of metabolites, with the enzymes responsible identified as camphor 5‐monooxygenase and ketolactonase I. A number of camphor‐degrading *Pseudomonas* species have been investigated, including *P. putida* G1, which initiated degradation with a cytochrome P450_cam_‐catalysed hydroxylation reaction (Eaton and Sandusky, 2009). Furthermore, evidence is emerging for the transformation of NAs in the environment by hydroxylation to generate oxy‐NAs of the type observed in the present study (Wang *et al.*, 2013; Lengger *et al.*, 2015). Although these metabolites were still present at day 33, they did not accumulate in proportion to the loss of the parent compound, suggesting that they would have continued to be transformed beyond 33 days and not accumulate as dead‐end metabolites. Nevertheless, it will be informative to assess the toxicity and biotransformation of these metabolites further.

### Change in toxicity during the biotransformation of the diamondoid carboxylic acids

In the present study, the toxicity of the diamondoid carboxylic acids decreased over time (as measured by Microtox^®^). Moreover, the transformation products were less toxic than the parent compounds. Our results showed that at day 0, enrichments containing A1CA were approximately 1.7‐fold more toxic based on the Microtox^®^ assay compared to 3EA based on EC_20_ (i.e. the median effective concentration which results in a 20% reduction and indicates a low level of acute toxicity). By day 33, the EC_20_ for both TPW and 2 m enrichment cultures increased 1.5‐2.0‐fold for A1CA and 3EA, suggesting that toxicity had reduced during biotransformation (Table 1).

**Table 1:** EC_20_ values as determined by Microtox^®^. The EC_20_ of the phenol standard was 3.95 mg l^−1^ (± 1.51).

Sample	Diamondoid carboxylic acid	
	EC_20_ A1CA (mg l^‐1^)^a^	EC_20_ 3EA (mg l^‐1^)^a^
TPW day 0	31.2 ± 2.1	52.7 ± 4.9
TPW day 33	49.5 ± 2.4	81.3 ± 5.2
2 m day 0	32.9 ± 2.1	46.7 ± 2.3
2 m day 33	57.1 ± 3.3	98.4 ± 4.2

Mean ± 95% confidence limits, *n = 3.*

Rowland *et al. *(2011c) also showed that the relative toxicity of alkylcyclohexylbutanoic acids was reduced by biotransformation to alkylcyclohexylethanoic acids, while Johnson *et al. *(2011) found the same reduction in toxicity for the corresponding aromatic acids. The predicted EC_50_ (extrapolated from EC_20_) for A1CA enrichments was 0.42 mM (at day 0) and 0.65 mM (at day 33), whilst 3EA enrichments the predicted EC_50_ (extrapolated from EC_20_) was 0.52 mM (at day 0) and 0.83 mM (at day 33) (Table 2). The maximum EC_50_ detected by Jones *et al. *(2011), using the Microtox^®^ assay, for diamondoid carboxylic acids was 0.337 mM for 3‐5‐dimethyl adamantane ethanoic acid, while the minimum EC_50_ was 0.784 mM for A1CA, suggesting that more‐branched diamondoid carboxylic acids are more toxic than the less‐branched compounds. These EC_50_ values are higher than those for other classes of NAs and related compounds tested by Jones *et al. *(2011) and Johnson *et al. *(2011) (Table 2). Frank *et al. *(2009) also showed that distilled fractions of oil sands acids had low EC_50_ values compared to individual acids, diamondoid or otherwise, perhaps demonstrating increased toxicity of these acids when present as complex mixtures that are typical of OSPW. Despite the relatively low acute toxicity, evidence of chronic toxicity of diamondoid carboxylic acids has been reported, with both A1CA and 3EA shown to disrupt human liver enzyme activity (Scarlett *et al.*, 2012). A1CA has also been found to have comparable genotoxicity to the widely studied polycyclic aromatic hydrocarbon (PAH), benzo[*a*]pyrene, in marine mussel toxicity assays (Dissanayake *et al.*, 2016) and demonstrable phytotoxicity against *Arabidopsis thaliana* (Leishman *et al.*, 2013).

**Table 2:** EC_50_ Microtox^®^ values for various NA compounds including predicted values for A1CA and 3EA from the present study.

Naphthenic acid compound	EC_50_ (mM) from Microtox^®^	References
A1CA	0.42	This study
3EA	0.52	This study
*n*‐acids	0.012–0.7	Jones *et al. *(2011)
Isoprenoid acids	0.015–0.24	Jones *et al. *(2011)
Monocyclic acids	0.012–0.41	Jones *et al. *(2011)
Monoaromatic acids	0.023–0.394	Jones *et al. *(2011)
Diamondoid acids	0.337–0.784	Jones *et al. *(2011)
Butylphenyl butanoic acids	0.05–0.21	Johnson *et al. *(2011)
Distilled fractions of oil sands acids	0.14–0.28	Frank *et al. *(2009)

### Characterisation of microbes putatively responsible for diamondoid carboxylic acid biotransformation

**Figure 4:**
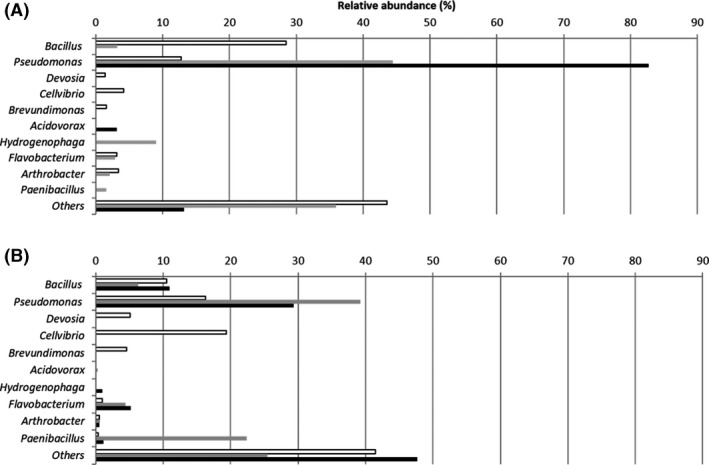
Bacterial communities from TPW enriched with A1CA and 3EA as sole source of carbon and energy and analysed by high‐throughput sequencing of the 16S rRNA gene. Bacterial genera compromising > 3% of the total community from Day 0 (white bars), Day 11 (grey bars) and Day 33 (black bars) in either A1CA (A) or 3EA (B) enriched communities. Genera representing < 3% of the community composition were assigned the classification ‘others’.

High‐throughput sequencing was performed to identify the main bacterial taxa that increased in relative abundance from one of the OSPW samples, TPW, when grown on each diamondoid carboxylic acid. A total of 22 operational taxonomic units (OTUs) (at the 95% identity level) were obtained from enrichments amended with A1CA or 3EA encompassing *Proteobacteria*, *Bacteroidetes*, *Actinobacteria* and *Firmicutes* (Fig. 4, Table S2). Indeed, OSPW are known to harbour a core microbiome including Betaproteobacteria, Gammaproteobacteria, Bacteroidetes and Firmicutes (Wilson *et al.*, 2016). The dominant OTUs present in A1CA‐amended enrichments (Fig 4A) at day 0 were *Bacillus* (28.5%) *Pseudomonas* (12.8%), *Cellvibrio* (4.2%), *Arthrobacter* (3.4%) and *Flavobacterium* (3.1%). By day 11, the relative abundance of *Pseudomonas* increased 3.5‐fold*. Hydrogenophaga* was the second most abundant genus by day 11 (9.0%). However, *Pseudomonas* remained the dominant genus by day 33, comprising 82.7% of the community, followed by *Acidovorax* (3.1%).

In the enrichments amended with 3EA (Fig. 4B), *Cellvibrio* (19.4%), *Pseudomonas* (16.2%), *Bacillus* (10.5%), *Devosia* (5.1%) and *Brevundimonas* (4.6%) were found at day 0. By day 11, *Pseudomonas* was dominant, comprising 39.2% of the sequences, followed by *Paenibacillus* (22.4%). Although the relative abundance of *Pseudomonas* decreased from day 11 to 29.3% at day 33, it remained the dominant genus. There was also an increase in the relative abundance of sequences from *Flavobacterium* during the enrichment (1% at day 0, 4.4% at day 11 and 5.2% at day 33). In both A1CA and 3EA enrichments, by day 33, *Pseudomonas* was the dominant genus. *Pseudomonas* species have been shown to dominate the microbial communities in other NA degradation studies, including the biodegradation of model aromatic NAs (Johnson *et al.*, 2011), commercial NAs (Del Rio *et al.*, 2006) and bitumen‐exposed river sediments (Yergeau *et al.*, 2012).

Eight bacterial isolates were obtained from enrichments amended with either A1CA or 3EA, which had a high 16S rRNA gene sequence identity to *Pseudomonas* spp., *Bacillus* spp., *Azotobacter* spp. and *Exiguobacterium* spp. (Table S3). Isolate 17, recovered from an enrichment culture inoculated with sample TPW and amended with A1CA, had a high (99%) 16S rRNA gene sequence identity to *Bacillus thuringiensis.* This isolate also had 99% 16S rRNA gene sequence identity to a *Bacillus* isolate previously shown to oxidise A1CA and a range of other NAs (Yue *et al.*, 2015). Three isolates, which were all obtained from enrichment cultures inoculated with sample 2 m and amended with A1CA, had 99% 16S rRNA gene sequence identity to *Pseudomonas guineae* (isolate 19), *Exiguobacterium aurantiacum* (isolate 20) and *Bacillus weihenstephanensis* (isolate 21). Whilst *Exiguobacterium* species have not previously been isolated from NA enrichments or NA‐contaminated environments, *Exiguobacterium‐*related sequences have been detected in OSPW sludge (Golby *et al.*, 2012) and OSPW bioreactors (McKenzie *et al.*, 2014). Isolates 23 and 24, derived from a TPW‐inoculated culture amended with 3EA, had 99% 16S rRNA gene sequence identity to *Pseudomonas balearica* and *Pseudomonas xanthomarina* respectively. In addition to the *Pseudomonas* strains that we isolated on diamondoid carboxylic acids, *P. fluorescens and P. putida* have also been isolated from enrichments amended with commercial NA mixtures (Del Rio *et al.*, 2006). Isolates 26 and 28, derived from 2 m‐inoculated and 3EA‐amended enrichments, had 99% 16S rRNA sequence identity to *Bacillus aquimaris* and *Azotobacter chroococcum* respectively. To date, this is the first study to isolate an *Azotobacter* species in relation to NA transformation or utilisation, although *Azotobacter* species have been shown to degrade chlorinated aromatic hydrocarbons (Anupama and Paul, 2009). In our study, no growth was observed with any of the isolates on agar plates with the same media but without the addition of the diamondoid carboxylic acid.

**Figure 5:**
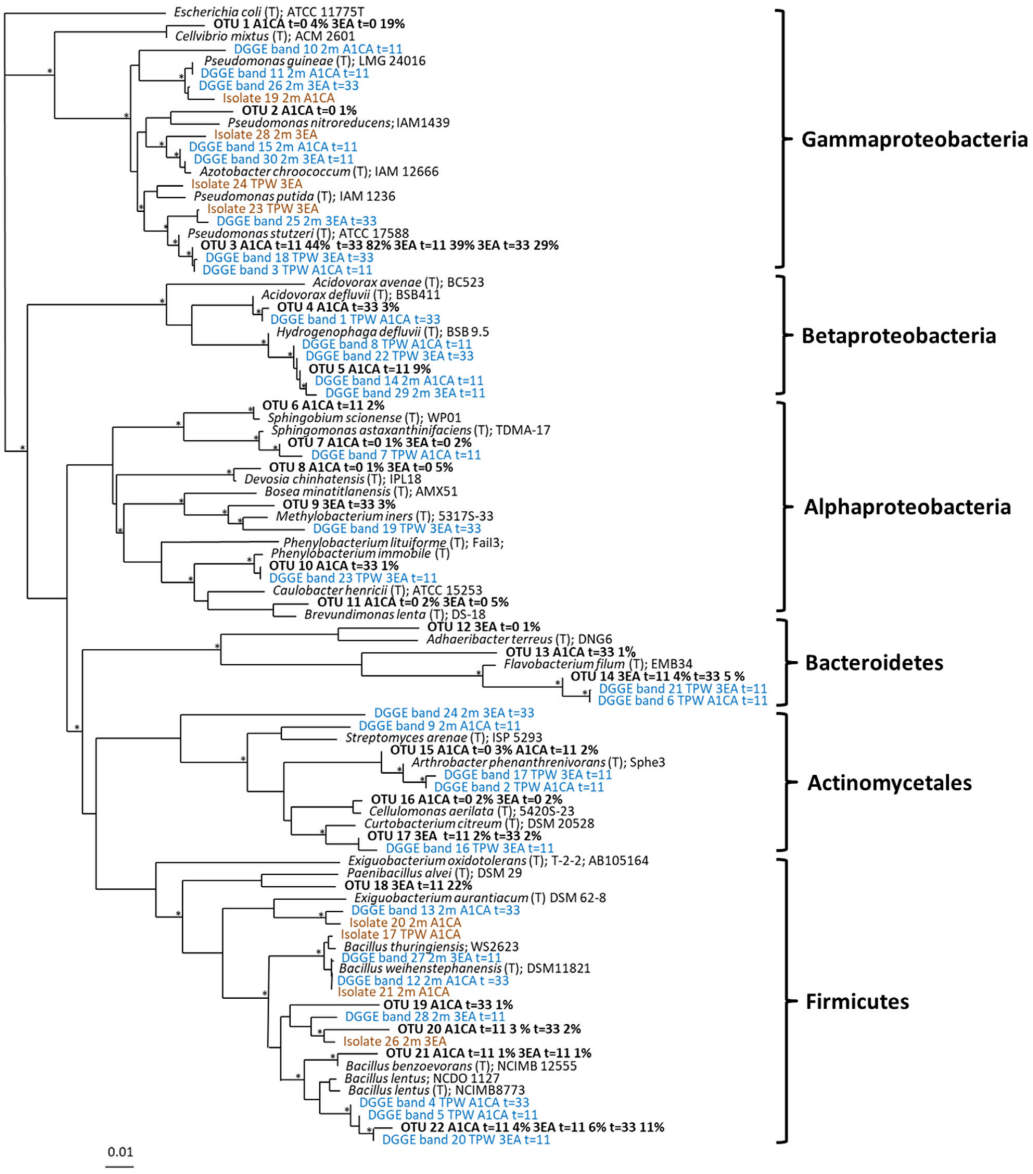
Bacterial phylogenetic tree based on 16S rRNA gene sequences, showing the relationship between sequences from a representative of the pyrosequence clusters, DGGE bands and isolates. DGGE bands (blue) isolates (brown) and pyrosequencing OTUs (bold typeface). Most closely related sequences from Genbank are also indicated. Sequence analysis was performed on 219 bp using the Neighbour‐joining method. Bootstrap values represent percentages from 100 replicates of the data and those of > 80% are shown by *. Scale bar indicates 0.01 substitutions per nucleotide. For each OTU % indicates percentage community composition, with time point (t = 0, day 0; t = 11, day 11 and t = 33, day 33) and compound (A1CA, adamantane‐1‐carboxylic acid, 3EA, 3‐ethyl adamantane carboxylic acid) information shown as appropriate.

Phylogenetic analysis allowed the comparison of 22 of the most abundant OTUs, 30 denaturing gradient gel electrophoresis (DGGE) bands and the eight bacterial isolates derived from enrichments with diamondoid carboxylic acids as sole source of carbon and energy (Fig. 5). Furthermore, OTU and isolate sequences were compared to sequences from uncultivated bacteria to investigate their potential environmental distribution (Table S5). Sequences grouped with known Actinobacteria, Firmicutes, Bacteroidetes, Alpha‐, Beta‐ or Gammaproteobacteria. Within the Gammaproteobacteria, sequences clustered with *Pseudomonas* species; notably, *P. stutzeri*, *P. guineae*, *P. nitroreducens* and *P. putida*, irrespective of diamondoid carboxylic acid amendment. Specifically, OTU 3, which dominated the communities at day 11 and day 33, clustered with *P. stutzeri.* OTU 3 also had 100% 16S rRNA gene sequence identity to nine clones (two from PAH‐contaminated soil, two from produced water and the rest from marine or freshwater environments) (Table S5). A number of DGGE bands that increased in relative intensity during enrichment also clustered with *Pseudomonas* spp. For example, Band 10 (from 2 m amended with A1CA, day 11), clustered with *P. guineae*; band 25 (from 2 m amended with 3EA, day 33) grouped with *P. putida*; whilst band 18 (amended with 3EA, day 11) and band 3 (amended with A1CA, day 11), both from TPW, clustered with *P. stutzeri* (Fig. S1, Table S4).

Within the Betaproteobacteria, several sequences grouped with *Hydrogenophaga* and *Acidovorax*, irrespective of inoculum or added diamondoid carboxylic acid*.* Specifically, OTU 4 (amended with A1CA, day 33) and DGGE band 1, which increased in relative band intensity by day 33 in TPW enrichments amended with A1CA, clustered with *Acidovorax defluvii*. OTU 4 also had 100% 16S rRNA gene sequence identity to 28 clones (27 from freshwater environments and one from oil‐field produced water (Table S5). OTU 5 (which comprised 9% of enrichments amended with A1CA at day 11) and DGGE band 22 (which increased in relative band intensity by day 33 in TPW enrichments amended with 3EA) clustered with *Hydrogenophaga defluvii* (Fig. S1, Table S4). OTU 5 also had 100% 16S rRNA gene sequence identity to 24 clones from a range of diverse environments, including five from hydrocarbon‐contaminated soil (Table S5). *Hydrogenophaga*, *Acidovorax* and *Flavobacterium* have also been identified previously in oil sands tailings (Ramos‐Padron *et al.*, 2011; Golby *et al.*, 2012; Yergeau *et al.*, 2012; An *et al.*, 2013), whilst *Acidovorax* have been detected in biofilms from OSPW (Golby *et al.*, 2012) and *Hydrogenophaga* shown to positively correlate with NA degradation in tailings ponds (Yergeau *et al.*, 2012). However, this is the first study to putatively associate *Hydrogenophaga, Acidovorax* and *Flavobacterium* with the biotransformation of diamondoid carboxylic acids.

Within the Firmicutes, OTU 18 which comprised 22% of the 3EA‐amended enrichments at day 11, grouped with *Paenibacillus alvei*. DGGE band 13, which increased in relative intensity at day 33 from A1CA‐amended 2 m enrichments along with isolate 20, clustered with *Exiguobacterium aurantiacum*. A number of sequences were also found to cluster with the Bacilli including OTUs 19‐22 and isolates 17, 21 and 26. DGGE bands 4 and 5 which increased in relative band intensity from TPW A1CA‐amended enrichments at days 11 and 33, respectively, grouped with *B. lentus* (Fig. S1, Table S4). *Bacillus* sequences have also been found to dominate NA‐degrading enrichments (Johnson *et al.*, 2011) and bacterial‐algal communities capable of degrading ~ 80% of A1CA, suggesting a possible role of these microorganisms in oil sands tailings pond remediation (Paulssen and Gieg, 2019). Overall, the present study implicates a range of taxa from the Gammaproteobacteria (in particular *Pseudomonas*), Betaproteobacteria and Firmicutes as key members of bacterial communities consuming diamondoid carboxylic acids, as found by others for the biodegradation of NAs.

### Pseudomonas stutzeri, a versatile and abundant member of the diamondoid carboxylic acid transforming communities

From our results, it is clear that *Pseudomonas* sequences increased in abundance during diamondoid carboxylic acid transformation irrespective of the diamondoid carboxylic acid, and various *Pseudomonas* spp. were isolated from both A1CA and 3EA enrichments. While *Pseudomonas* spp. have not been previously linked with diamondoid carboxylic acid biotransformation, in our study, *Pseudomonas* spp. dominated the enrichments. Specifically, one dominant OTU (OTU 3), which constituted 82% of the sequences from A1CA and 29% from 3EA‐amended enrichments at day 33, as well as Isolate 23 which grew on 3EA as sole source of carbon and energy, was closely related to *P. stutzeri.* Thus, this broad and versatile diazotrophic species that can grow aerobically or with nitrate as a terminal electron acceptor (Lalucat *et al.*, 2006) is likely to play an important role in diamondoid carboxylic acid biotransformation. Herman *et al. *(1994) also isolated a strain of *P. stutzeri* from enrichments containing environmental NAs, and *P. stutzeri* has also been shown to degrade the PAHs: naphthalene (Brunet‐Galmés *et al.*, 2012), phenanthrene (Grimberg *et al.*, 1996), pyrene (McNally *et al.*, 1999) and a wide range of other hydrocarbons, heterocyclic compounds and chlorinated and carboxylated derivatives of hydrocarbons (Lalucat *et al.*, 2006). *P. stutzeri* was also relatively abundant at day 0, indicating that it may play an important role in degradation of NA and other compounds in the OSPW. Indeed, *P. stutzeri* was enriched on high molecular weight PAHs from the same sample, TPW, by Folwell *et al. *(2016), and has also been detected in abundance in a range of hydrocarbon‐contaminated environments, including a tar pit (Kim and Crowley, 2007) and oil reservoirs (Zhang *et al.*, 2014). Therefore, the capacity to transform structurally complex diamondoid carboxylic acids as well as a range of other toxic compounds found is OSPW, makes *P. stutzeri* a prime candidate for bioremediation of such environments.

## Conclusions

In conclusion, microbial communities isolated from two samples of OSPW have been shown to aerobically transform the diamondoid carboxylic acids, A1CA and 3EA, over 33 days while the toxicity of the compounds was reduced. During transformation, two hydroxyl‐metabolites were putatively identified as 2‐hydroxyadamantane‐1‐carboxylic acid (from A1CA) and 3‐ethyladamantane‐2‐ol (from 3EA). It is probable that some microorganisms in the enrichments were conserving energy for growth from diamondoid NAs based on the following evidence: (i) the metabolites did not accumulate in proportion to the loss of the parent compounds and no other peaks were detected, suggesting further degradation of the metabolites; (ii) diamondoid carboxylic acids were the sole source of carbon and energy supplied; and (iii) taxa that increased in relative abundance in enrichments frequently corresponded with those that were isolated and also shown to grow when supplied with diamondoid carboxylic acids. Differences in the day‐33 TPW community suggest that diverse microbial taxa are involved in the transformation of A1CA and 3EA with *Pseudomonas* spp., notably *P. stutzeri,* playing a key role in diamondoid carboxylic acid transformations. The identification of microorganisms capable of transforming these diamondoid carboxylic acids under aerobic conditions from OSPW contributes to ongoing efforts to remediate OSPW by aeration. Future challenges will be to identify and quantify the NAs that are contributing to the toxicity of OSPW in order to validate targets for bioremediation studies.

## Experimental procedures

### Samples used in this study

A surface water sample (designated TPW, pH 7.44) was collected from a Syncrude test pit (GIS 57.0380885, −111.5407123) courtesy of Warren Zubot (Syncrude, Wood Buffalo, Alberta, Canada). The test pit was excavated in 1993 and filled with aged recycled water from the Mildred Lake Settling Basin (MLSB). A second water sample (designated 2 m, pH 7.68) was supplied by Lisa Gieg (University of Calgary) and collected at a water depth of 2 m from a Suncor tailings pond (GIS 56.9923073, −111.4979146). Ion chromatography, using an ICS – 3000 Dionex, was performed as previously described (Folwell *et al.*, 2016), and the anion and cation profiles are given in Table S1. Total organic carbon (TOC) analysis was performed using a Shimadzu TOC‐VCHS pyrolyser fitted with a SSM 5000A solid sample module and running TOC‐Control V software v. 2.00. The TOC for TPW was 28.8 g l^−1^ and for 2 m 24.3 g l^−1^.

### Diamondoid carboxylic acid biotransformation experiment

The minimal medium used throughout contained per litre: MgSO_4_.7H_2_O, 0.5 g; CaCl_2._H_2_O, 0.1 g; NH_4_NO_3,_ 1 g; Na_2_HPO_4,_ 1.1 g; KH_2_PO_4_, 0.25 g, trace elements, 1 ml, (which contained per litre: FeSO_4_.7H_2_O; 10 mg; Na_2_EDTA.3H_2_O, 0.64 mg; ZnSO_4_.7H_2_O, 0.2 mg; H_3_BO_3_, 0.015 mg; CoCl_2_.6H_2_O, 0.175 mg; Na_2_MoO_4_, 0.14 mg; MnCl_2_.4H_2_O, 0.02 mg; NiCl_2_.6H_2_O, 0.01 mg adjusted to pH 7.0 and autoclaved) (Folwell *et al.*, 2016). For isolation of colonies, the growth medium also contained 15 g l^−1^ of bacteriological agar (Difco), in addition to the diamondoid carboxylic acids (5 mg l^−1^ final concentration). Serum bottles were set up containing 100 ml minimal media and 1% (v/v) inoculum of either TPW or 2 m. Enrichments were amended with either 5 mg l^−1^ of adamantane‐1‐carboxylic acid (A1CA) or 3‐ethyladamantane‐carboxylic acid (3EA) (obtained from Sigma‐Aldrich, Gillingham UK, HPLC grade, >98% purity for both). Serum bottles were capped, crimp sealed and incubated aerobically at 20°C. Killed controls were also prepared by autoclaving cultures three times and incubating at 20°C overnight between autoclave cycles before carboxylic acid addition. Viability was checked by inoculating 100 µl of culture on LB plates and incubating at 20°C. Abiotic controls containing the carboxylic acids and procedural blanks of un‐inoculated minimal media supplemented with the A1CA, 3EA (at 5 mg l^−1^) were also prepared. A 25 ml of sub‐sample was taken at day 0, 11 and 33 and centrifuged at 3435 *g* for 10 min. The supernatant and cell pellets were separated and stored at −20°C.

### Diamondoid carboxylic acid extraction and GC‐MS analysis

All glassware was prepared as described previously (Johnson *et al.*, 2011). The internal standard was 4‐phenylbutanoic acid (Acros Organics) (10 mg) dissolved in 0.1 M NaOH (final concentration 2 mg l^−1^) and added to each culture immediately prior to extraction. Samples were acidified to pH 2 using concentrated HCl, and the diamondoid carboxylic acids were extracted from the supernatants three times using 15 ml of ethyl acetate (HPLC grade; Fisher Scientific, Loughborough, UK) as previously described (Johnson *et al.*, 2011). Solvent extracts were pooled, dried with 5–10 g anhydrous Na_2_SO_4_ (Fisher Scientific), and concentrated by rotary evaporation (Buchi) at 40°C. Samples were transferred to a gas chromatography vial (Chromacol) and stored at −20°C. Samples were injected with a 1 µl splitless injection (injector temperature 250°C) onto a 30 m x 250 µm x 0.25 µm Rtx – 1MS column using helium as the carrier gas at a constant flow of 1 ml min^‐1^. Oven temperatures were as follows: an increase from 40 to 250°C at 10°C min^‐1^ followed by a final hold at 250°C for 10 min. The transfer line was held at 230°C onto a source for the MS which was in full‐scan mode (scan range 50–650 Da). Data were analysed and integrated using ChemStation for GC‐MS (Agilent, Stockport, UK).

### Microtox analysis^®^

The toxicity of A1CA and 3EA was measured by Microtox^®^ assay (Azur Environmental, Carlsbad, CA, USA) using the 81.9% basic liquid‐phase test protocol as described by the manufacturer. Triplicate samples from day 0 and 33 were added to reconstituted cells, and 18 mg l^−1^ phenol was used as a standard. Exposure times of 15 min are reported.

### PCR‐DGGE analysis of Bacterial 16S rRNA genes

DNA was extracted from cell pellets (at days 0, 11 and 33) as previously described (Grabowski *et al.*, 2005). All PCR amplifications were performed using a Gene Amp^®^ PCR system 9700 Thermocycler (Applied Biosystems, Warrington, UK), and amplification products were analysed using a 1% (w/v) agarose gel in 1× TAE (40 mM Tris base, 1 mM EDTA [pH 8]), stained with ethidium bromide (10 mg ml^−1^) and viewed under UV transillumination (Alpha Innotech, Exeter, UK). PCR‐DGGE analysis was performed using bacterial primers F341‐GC and R534 and cycling conditions as previously described (Muyzer *et al.*, 1993). DGGE analysis was performed using a D‐Code System (Bio‐Rad, Watford, UK) as described by Muyzer *et al. *(1993), with a gradient of 40–60%, except that gels were silver stained as described elsewhere (Acuña Alvarez *et al.*, 2009). Selected DGGE bands were excised and placed into 100 µl of nuclease‐free water for storage at 4°C. DGGE bands were re‐amplified using the F341 and R534 primers for bacterial DNA, purified using GenElute^TM^ PCR Clean‐Up Kit (Sigma‐Aldrich) and sequenced by Source Bioscience (UK). The sequences obtained were compared with public DNA database sequences using Basic Local Alignment Search Tool (BLAST*n*).

### High‐throughput sequencing

The bacterial 16S rRNA gene from TPW enrichments with both diamondoid carboxylic acids at days, 0, 11 and 33 was sequenced using 454 pyrosequencing. The 16S rRNA gene was amplified using the primers Bakt_341F (CCTACGGGNGGCWGCAG) and Bakt_805R (GACTACHVGGGTATCTAATCC) (Herlemann *et al.*, 2011). GS FLX Titanium adaptors were at the 5′‐end of the Bakt primers, adaptor A for the forward primer (5′‐CGTATCGCCTCCCTCGCGCCATCAG‐3′) and B for the reverse primer (5'‐CTATGCGCCTTGCCAGCCCGCTCAG‐3′). Each sample was given a unique reverse 10 bp primer barcode located between the B adaptor and Bakt_805R as described by Aslam *et al. *(2016). Cycling conditions were as described in Herlemann *et al. *(2011). Triplicate PCR products were pooled, purified using a QIAquick Gel Extraction kit (Qiagen, Manchester, UK) and analysed using the FLX 454 Titanium sequencer (Roche, Welwyn Garden City, UK) at Wageningen University (The Netherlands). Reads were analysed using the QIIME pipeline and associated modules as described by Folwell *et al. *(2016). All sequences were checked for the presence of correct sequencing adaptors, 10 base barcodes and the 16S rRNA gene‐specific primers, and any sequences containing errors in these regions were removed. Any sequences < 150 bp in read length, containing 7 bp homopolymer inserts, with low‐quality scores (< 25) or chimeras were also removed. The remaining reads were clustered into operational taxonomic units (OTUs) using the USearch algorithm at the 95% similarity level (Edgar, 2010). Representative sequences from each OTU were assigned to a taxonomic group using the RDP classifier algorithm (Wang *et al.*, 2007) and their percentage relative abundance calculated. For phylogenetic analysis, sequences were aligned with selected sequences from the Genbank database using the RDP INFERNAL alignment tool (Nawrocki and Eddy, 2007). Alignments were checked using ClustalX, and phylogenetic analysis was performed using PHYLIP 3.6 with Jukes‐Cantor (Jukes and Cantor, 1969) and neighbour‐joining methods (Saitou and Nei, 1987). Bootstrap analysis was based on 1000 replicates using SEQBOOT (PHYLIP 3.6). Tree construction was performed using TreeView (Win 32) 1.6.6 (Page, 1996).

### Isolation and identification of pure cultures

For each diamondoid carboxylic acid (A1CA and 3EA), 10 mg was dissolved in 1 ml of ethyl acetate (Fisher Scientific), 100 μl of which was spiked onto a washed minimal media agar plate as described (Johnson *et al.*, 2012). Prior to inoculation, plates were left for 1 h for the ethyl acetate to evaporate. Prior to centrifugation, a sample (100 µl) was taken from enrichment cultures and inoculated onto the agar plates containing either A1CA or 3EA as sole source of carbon and energy. Plates were incubated at 20°C for 10 days. Single colonies were selected and sub‐cultured up to five times on plates containing the target diamondoid carboxylic acid until pure colonies were obtained. Samples were also inoculated on to minimal media agar plates containing no added diamondoid carboxylic acids to identify preferential growth in the presence of A1CA or 3EA. DNA was extracted from isolates as previously described (Grabowski *et al.*, 2005). PCR amplification was performed using primers 27F and 1492R (Turner *et al.*, 1999). Each 50 µl PCR mixture contained 50–100 ng DNA, primers (0.4 µM), dNTPs (0.1 mM), *Taq* polymerase (1.25 U; Qiagen) and 1x PCR buffer (Qiagen) with the following cycling conditions: 94°C for 5 min followed by 30 cycles of 94°C for 30 s, 57°C for 45 s, 72°C for 90 s, with 72°C for 10 min. The amplified 16S rRNA genes were Sanger sequenced at Source Bioscience, UK.

### Statistical analysis

All statistical analyses were performed using SPSS version 18.0 (IBM United Kingdom Limited, Portsmouth, UK) and Primer 6 Beta (Quest Research Limited, Auckland, New Zealand) using standard tests. For all biotransformation studies, statistical analysis was performed using an exact Mann–Whitney *U* test. The two‐tailed significance was reported.

## Conflict of interest

None declared.

## Author contributions

T.J.M. and C.W conceived the idea and wrote the original proposal to obtain the funding for the PhD studentship. T.J.M and C.W. conceived and designed the experiments with input from B.D.F. B.D.F. conducted all the experimental work and analysed the experimental data. T.J.M, C.W. and B.D.F. all prepared the manuscript.

## Supporting information


**Fig. S1**. DGGE analysis of enrichments TPW and 2 m, demonstrating microbial community structure change under the selective pressure of diamondoid carboxylic acids. Transformation of A1CA over 33 days by TPW (A) and 2 m (C) and of 3EA by TPW (B) and 2 m (D) communities. Numbers refer to bands identified in Table S2.
**Table S1**. Anion and cation analysis of TPW and 2 m OSPW samples (ND: Not detected).
**Table S2**. Composition of bacterial communities in A1CA and 3EA transforming enrichments with TPW as inoculum (day 0, 11 and 33) based on high‐throughput sequencing analysis of 16S rRNA genes. Note that when the same genus is mentioned more than once it indicates different OTUs from the same genus.
**Table S3**. Blast*n* analysis of 16S rRNA sequences from DGGE bands excised from the A1CA and 3EA transforming communities derived from samples TPW and 2 m.
**Table S4**. BLAST*n* analysis of the 16S rRNA gene sequences obtained from isolates.
**Table S5**. Source and distribution of environmental sequences with >97% 16S rRNA gene sequence identity to the operational taxonomic units (OTUs) obtained from this study.
