# Supplementary material for: Diamondoids are not forever: microbial biotransformation of diamondoid carboxylic acids
Source: Microb Biotechnol. 2019 Nov 12;13(2):495–508. doi: 10.1111/1751-7915.13500 (PMC7017837; doi:10.1111/1751-7915.13500)
Supplement: Supplementary file 1 — Fig. S1. DGGE analysis of enrichments TPW and 2 m, demonstrating microbial community structure change under the selective pressure of diamondoid carboxylic acids. Transformation of A1CA over 33 days by TPW (A) and 2 m (C) and of 3EA by TPW (B) and 2 m (D) communities. Numbers refer to bands identified in Table S2. Table S1. Anion and cation analysis of TPW and 2 m OSPW samples (ND: Not detected). Table S2. Composition of bacterial communities in A1CA and 3EA transforming enrichments with TPW as inoculum (day 0, 11 and 33) based on high‐throughput sequencing analysis of 16S rRNA genes. Note that when the same genus is mentioned more than once it indicates different OTUs from the same genus. Table S3. Blastn analysis of 16S rRNA sequences from DGGE bands excised from the A1CA and 3EA transforming communities derived from samples TPW and 2 m. Table S4. BLASTn analysis of the 16S rRNA gene sequences obtained from isolates. Table S5. Source and distribution of environmental sequences with >97% 16S rRNA gene sequence identity to the operational taxonomic units (OTUs) obtained from this study. [file MBT2-13-495-s001.docx]

**Supplementary Data for Review**

Ion chromatography, using an ICS – 3000 Dionex was performed as previously described (Folwell *et al.,* 2016) and the anion and cation profiles are given (**Table S1**).

**16S rRNA gene sequence analysis of bacterial communities, DGGE bands and bacterial isolates.**

The composition of bacterial communities in A1CA and 3EA transforming enrichments with TPW as the inoculum (day 0, 11 and 33) based on high throughput sequencing analysis of 16S rRNA genes was performed. **Table S2** presents a detailed summary of the genera comprising >1% of the total bacterial community. Note that when the same genus is mentioned more than once it indicates different OTUs from the same genus.

DGGE was performed to provide a broad comparison of the differences in the main taxa between treatments (**Fig. S1**). DGGE bands 1–30 were sequenced and the closest BLAST*n* match presented (**Table S3)**. DGGE bands 3 and 18 had high 16S rRNA gene sequence identity to *Pseudomonas stutzeri* (99%) and were present in A1CA and 3EA enriched TPW communities, respectively. Similarly, DGGE bands 4 and 20, which had high 16S rRNA gene sequence identity to *Bacillus lentus* (98%), were present in both A1CA and 3EA enriched TPW communities. DGGE bands 8, 14, 22 and 29 all had high 16S rRNA gene sequence identity to *Hydrogenophaga* spp. (98-99%), and were present in all communities regardless of inoculum or substrate. 16S rRNA gene sequences was also performed from eight bacterial isolates obtained from enrichments amended with either A1CA or 3EA (**Table S4**) and showed high sequence identity to *Pseudomonas* spp., *Bacillus* spp., *Azotobacter* spp. and *Exiguobacterium* spp.

**Fig S1.**


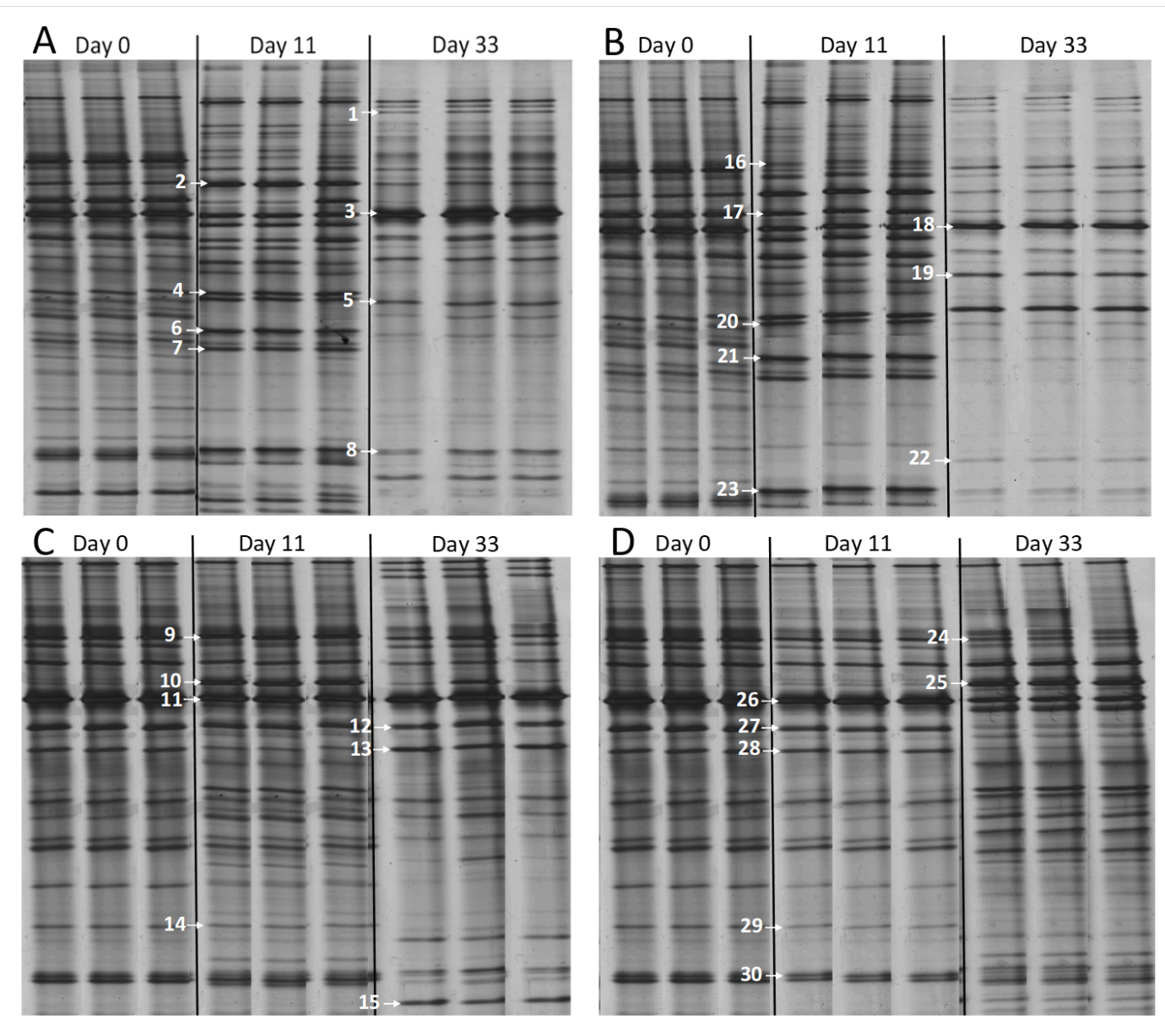


**Table S1**

| **Ion** | **Concentration in OSPW TPW sample (µmol L^-1^)** | **Concentration in OSPW 2m sample (µmol L^-1^)** |
| --- | --- | --- |
| Fluoride | 131.3 | 490 |
| Formate | 14.2 | 349.3 |
| Acetate | ND | ND |
| Chloride | 7164.5 | 14334.9 |
| Bromide | 3.3 | 9.1 |
| Nitrate | 2.4 | 14 |
| Sulfate | 1264 | 1419.5 |
| Phosphate | ND | 18 |
| Lithium | 45.1 | 121.2 |
| Sodium | 28290.7 | 36136.6 |
| Potassium | 475 | 3479.3 |
| Magnesium | 728.9 | 1970 |
| Calcium | 512.6 | 2174.3 |
| Strontium | 11.5 | 13.4 |
| Barium | 10.32 | 94.5 |
|  |  |  |

**Table S2**

| **Enrichment** | | | **A1CA Day 0** | **A1CA Day 11** | **A1CA Day 33** | **3EA**  **Day 0** | **3EA Day 11** | **3EA Day 33** |
| --- | --- | --- | --- | --- | --- | --- | --- | --- |
| **Number of sequences retrieved** | | | 235 | 369 | 2967 | 1028 | 800 | 810 |
| **Class** | **Genus** | **OTU** | **Percentage of community** | | | | | |
|  |  |  |  |  |  |  |  |  |
| Gammaproteobacteria | *Cellvibrio* | *1* | **4.2** | 0 | 0 | **19.4** | 0 | 0.7 |
|  | *Pseudomonas* | *2* | 1.1 | 0 | 0 | 0.1 | 0.2 | 0.4 |
|  | *Pseudomonas* | *3* | **12.8** | **44.4** | **82.7** | **16.2** | **39.2** | **29.3** |
| Betaproteobacteria | *Acidovorax* | *4* | 0 | 0.8 | **3.1** | 0 | 0.2 | 0 |
|  | *Hydrogenophaga* | *5* | 0 | **9.0** | 0 | 0.8 | 0.2 | 1.2 |
| Alphaproteobacteria | *Sphingobium* | *6* | 0 | 1.6 | 0 | 1.1 | 0.6 | 0.1 |
|  | *Sphingomonas* | *7* | 1.2 | 0 | 0.7 | 1.8 | 0.6 | 0.1 |
|  | *Devosia* | *8* | 1.4 | 0.8 | 0 | **5.1** | 0 | 0.7 |
|  | *Microvirga* | *9* | 0.8 | 0 | 0.2 | 1.9 | 1.6 | 2.9 |
|  | *Phenylobacterium* | *10* | 0 | 0 | 0.9 | 0.2 | 0 | 0.2 |
|  | *Brevundimonas* | *11* | 1.6 | 1.1 | 0.2 | **4.6** | 0.2 | 0.1 |
| Bacteroidetes | *Adhaeribacter* | *12* | 0 | 0 | 0.1 | 1.1 | 0.4 | 0.2 |
|  | *Lutibacter* | *13* | 0 | 0 | 1.1 | 0 | 0 | 0 |
|  | *Flavobacterium* | *14* | **3.1** | 2.9 | 0 | 1 | **4.4** | **5.2** |
| Actinomycetales | *Arthrobacter* | *15* | **3.4** | **2.1** | 0.1 | 0.5 | 0.6 | 0.5 |
|  | *Cellulomonas* | *16* | 1.7 | 0.2 | 0.1 | 1.6 | 0.2 | 0.5 |
|  | *Curtobacterium* | *17* | 0.8 | 0 | 0.2 | 1.9 | 1.6 | 1.8 |
| Firmicutes | *Paenibacillus* | *18* | 0 | 1.6 | 0.1 | 0.4 | **22.4** | 1.1 |
|  | *Planococcus* | *19* | 0 | 0 | 1.1 | 0 | 0 | 0 |
|  | *Bacillus* | *20* | 0 | 2.7 | 1.6 | 0 | 0.1 | 0.1 |
|  | *Bacillus* | *21* | 0 | 1 | 0.1 | 0.4 | 1.1 | 0.3 |
|  | *Bacillus* | *22* | **28.5** | **4.1** | 0.5 | **10.5** | **6.3** | **10.9** |
| Others | - | - | **39.4** | **27.7** | **7.2** | **31.4** | **20.1** | **43.7** |

**Table S3**

| **DGGE band** | **Closest match from Blast*n*** | **Sequence length**  **(bp)** | **% 16S rRNA gene sequence identity** | **Environment from which closest match was derived** | **Genbank accession number of most similar sequence** |
| --- | --- | --- | --- | --- | --- |
| **TPW sample amended with A1CA** | | | | | |
| 1 | *Acidovorax* spp. | 182 | 99 | Activated sludge | [NR_026506.1](http://www.ncbi.nlm.nih.gov/nucleotide/219846914?report=genbank&log$=nucltop&blast_rank=1&RID=750FHHFY01R) |
| 2 | *Arthrobacter* spp. | 209 | 99 | Soil | [NR_074770.1](http://www.ncbi.nlm.nih.gov/nucleotide/444439455?report=genbank&log$=nucltop&blast_rank=3&RID=750HUN4G01R) |
| 3 | *Pseudomonas stutzeri* | 183 | 99 | Sol | [NR_103934.1](http://www.ncbi.nlm.nih.gov/nucleotide/526641937?report=genbank&log$=nucltop&blast_rank=1&RID=750MGDAU01R) |
| 4 | *Bacillus lentus* | 192 | 98 | Soil | [NR_040792.1](http://www.ncbi.nlm.nih.gov/nucleotide/343200105?report=genbank&log$=nucltop&blast_rank=1&RID=750VAUPJ01R) |
| 5 | *Bacillus* *methanolicus* | 203 | 98 | Soil | [NR_040985.1](http://www.ncbi.nlm.nih.gov/nucleotide/343200298?report=genbank&log$=nucltop&blast_rank=1&RID=750PZ1S7015) |
| 6 | *Flavobacterium* spp*.* | 188 | 96 | Activated sludge | [NR_043767.1](http://www.ncbi.nlm.nih.gov/nucleotide/343204344?report=genbank&log$=nucltop&blast_rank=1&RID=75111JTC014) |
| 7 | *Sphingomonas astaxanthinifaciens* | 196 | 98 | Freshwater | [NR_041535.1](http://www.ncbi.nlm.nih.gov/nucleotide/343200848?report=genbank&log$=nucltop&blast_rank=1&RID=A12UU82J014) |
| 8 | *Hydrogenophaga* spp. | 191 | 99 | Activated sludge | [NR_029024.1](http://www.ncbi.nlm.nih.gov/nucleotide/265678719?report=genbank&log$=nucltop&blast_rank=1&RID=7512X9JS01R) |
| **2m sample amended with A1CA** | | | | | |
| 9 | *Streptomyces violaceus* | 185 | 98 | Soil | [NR_041115.1](http://www.ncbi.nlm.nih.gov/nucleotide/343200428?report=genbank&log$=nucltop&blast_rank=51&RID=751VG43001R) |
| 10 | *Pseudomonas denitrificans* | 198 | 99 | Soil | [NR_102805.1](http://www.ncbi.nlm.nih.gov/nucleotide/507147998?report=genbank&log$=nucltop&blast_rank=4&RID=751FT83F015) |
| 11 | *Pseudomonas guineae* | 179 | 97 | Antarctic | [NR_042607.1](http://www.ncbi.nlm.nih.gov/nucleotide/343202321?report=genbank&log$=nucltop&blast_rank=1&RID=6Z5UX1FD014) |
| 12 | *Bacillus weihenstephanensis* | 205 | 99 | Soil | [NR_074926.1](http://www.ncbi.nlm.nih.gov/nucleotide/444439611?report=genbank&log$=nucltop&blast_rank=4&RID=751DCCSJ01R) |
| 13 | *Exiguobacterium aurantiacum* | 202 | 99 | Permafrost | [NR_043478.1](http://www.ncbi.nlm.nih.gov/nucleotide/343202975?report=genbank&log$=nucltop&blast_rank=1&RID=751BRB4N01R) |
| 14 | *Hydrogenophaga* spp. | 191 | 98 | Activated sludge | [NR_029023.1](http://www.ncbi.nlm.nih.gov/nucleotide/265678718?report=genbank&log$=nucltop&blast_rank=2&RID=7512X9JS01R) |
| 15 | *Azotobacter* spp. | 189 | 97 | Soil | [NR_041035.1](http://www.ncbi.nlm.nih.gov/nucleotide/343200348?report=genbank&log$=nucltop&blast_rank=48&RID=751NBJ1301R) |
| **TPW sample amended with 3EA** | | | | | |
| 16 | *Curtobacterium* spp. | 195 | 98 | Soil | [NR_104839.1](http://www.ncbi.nlm.nih.gov/nucleotide/559795249?report=genbank&log$=nucltop&blast_rank=2&RID=A18CZBSN014) |
| 17 | *Arthrobacter* spp. | 207 | 99 | Soil | [NR_074770.1](http://www.ncbi.nlm.nih.gov/nucleotide/444439455?report=genbank&log$=nucltop&blast_rank=3&RID=750HUN4G01R) |
|  |  |  |  |  |  |
| 18 | *Pseudomonas stutzeri* | 186 | 99 | Soil | [NR_103934.1](http://www.ncbi.nlm.nih.gov/nucleotide/526641937?report=genbank&log$=nucltop&blast_rank=1&RID=750MGDAU01R) |
| 19 | *Methylobacterium* spp. | 190 | 95 | Soil | [NR_044129.1](http://www.ncbi.nlm.nih.gov/nucleotide/343205726?report=genbank&log$=nucltop&blast_rank=3&RID=750XJFCA014) |
| 20 | *Bacillus lentus* | 207 | 98 | Soil | [NR_040792.1](http://www.ncbi.nlm.nih.gov/nucleotide/343200105?report=genbank&log$=nucltop&blast_rank=1&RID=750VAUPJ01R) |
| 21 | *Flavobacterium* spp. | 219 | 96 | Activated sludge | [NR_043767.1](http://www.ncbi.nlm.nih.gov/nucleotide/343204344?report=genbank&log$=nucltop&blast_rank=1&RID=75111JTC014) |
| 22 | *Hydrogenophaga* spp. | 204 | 99 | Activated sludge | [NR_029024.1](http://www.ncbi.nlm.nih.gov/nucleotide/265678719?report=genbank&log$=nucltop&blast_rank=1&RID=7512X9JS01R) |
| 23 | *Phenylobacterium* spp. | 189 | 98 | Aquifer | [NR_029117.1](http://www.ncbi.nlm.nih.gov/nucleotide/265678812?report=genbank&log$=nucltop&blast_rank=1&RID=A14J1NDE01R) |
| **2m sample amended with 3EA** | | | | | |
| 24 | *Streptomyces* spp. | 195 | 98 | Soil | [NR_041115.1](http://www.ncbi.nlm.nih.gov/nucleotide/343200428?report=genbank&log$=nucltop&blast_rank=51&RID=751VG43001R) |
| 25 | *Pseudomonas balearica* | 205 | 99 | Soil | [NR_025972.1](http://www.ncbi.nlm.nih.gov/nucleotide/219846381?report=genbank&log$=nucltop&blast_rank=27&RID=751NBJ1301R) |
| 26 | *Pseudomonas guineae* | 188 | 97 | Soil | [NR_042607.1](http://www.ncbi.nlm.nih.gov/nucleotide/343202321?report=genbank&log$=nucltop&blast_rank=1&RID=6Z5UX1FD014) |
| 27 | *Bacillus weihenstephanensis* | 219 | 99 | Soil | [NR_074926.1](http://www.ncbi.nlm.nih.gov/nucleotide/444439611?report=genbank&log$=nucltop&blast_rank=4&RID=751SYGM701R) |
| 28 | *Bacillus aqumaris* | 201 | 97 | Sediment | [NR_025241.1](http://www.ncbi.nlm.nih.gov/nucleotide/219857652?report=genbank&log$=nucltop&blast_rank=9&RID=750PZ1S7015) |
| 29 | *Hydrogenophaga* spp. | 181 | 98 | Activated sludge | [NR_029023.1](http://www.ncbi.nlm.nih.gov/nucleotide/265678718?report=genbank&log$=nucltop&blast_rank=2&RID=7512X9JS01R) |
| 30 | *Azotobacter* spp. | 207 | 97 | Soil | [NR_041035.1](http://www.ncbi.nlm.nih.gov/nucleotide/343200348?report=genbank&log$=nucltop&blast_rank=48&RID=751NBJ1301R) |

| **Isolate ID** | **Carboxylic acid** | **Inoculum** | **Closest match from BLAST*n*** | **Sequence length**  **(bp)** | **% 16S rRNA gene sequence identity** | **Genbank accession number of most similar sequence** |
| --- | --- | --- | --- | --- | --- | --- |
| 17 | A1CA | TPW | *Bacillus thuringiensis* | 898 | 99 | KX150804.1 |
| 19 | A1CA | 2m | *Pseudomonas guineae* | 880 | 99 | NR042607.1 |
| 20 | A1CA | 2m | *Exiguobacterium aurantiacum* | 912 | 99 | NR113666.1 |
| 21 | A1CA | 2m | *Bacillus weihenstephanensis* | 910 | 99 | NR074926.1 |
| 23 | 3EA | TPW | *Pseudomonas balearica* | 876 | 99 | NR025972.1 |
| 24 | 3EA | TPW | *Pseudomonas xanthomarina* | 918 | 99 | NR041044.1 |
| 26 | 3EA | 2m | *Bacillus aquimaris* | 902 | 99 | NR025241.1 |
| 28 | 3EA | 2m | *Azotobacter chroococcum* | 890 | 99 | NR116305.1 |

**Table S4**

**Table S5**

| **OTU from this Study** | **Number of matches** | **Closest match accession number** | **% identity**  **match** | **Comments** |
| --- | --- | --- | --- | --- |
| OTU 3 | 9 | - | 100 | Of the 9 clones 2 were from PAH contaminated soil, 2 from oil field produced water with remainder from other marine (2) or freshwater environments (3) |
| OTU 4 | 28 | - | 100 | The majority of clones were from freshwater environments (27), with 1 sequence from oil field produced water |
| OTU 5 | 24 | - | 100 | Clones were found from a diverse range of environments, including freshwater (8), groundwater (6), hydrocarbon contaminated soil (5) and marine (3) |
| OTU 14 | 3 |  | 99.75 | Of the 3 clones, 1 was from a freshwater environment and 2 from groundwater environments |
| OTU 18 | 1 | KC620702 | 97.28 | The single clone with the highest % ID was from a groundwater environment |
| OTU 19 | 1 | AB929620 | 99.25 | The single clone with the highest % ID was from a freshwater environment |
| OTU 20 | 14 | - | 99.75 | The majority of clones were from soil (11), with 1 sequence from hydrocarbon contaminated soil |
| OTU 21 | 13 | - | 100 | No information available. All closest matches listed as environmental sample only |
| OTU 22 | 1 | AB637247 | 98.75 | No information available. Closest match listed as environmental sample only |
| Isolate 17 | 1 | KC466213 | 99.89 | No information available. Closest match listed as environmental sample only |
| Isolate 19 | 2 | - | 99.9 | The two clones were from groundwater environments |
| Isolate 20 | 2 | - | 99.81 | Of the two clones, 1 was from a marine environment and 1 from a freshwater environment |
| Isolate 21 | 6 | - | 99.79 | Of the 6 clones, 5 were from soil and 1 from groundwater |
| Isolate 23 | 3 | - | 99.28 | Of the 3 clones, 1 was from an oil field, 1 from a marine environment and 1 from a groundwater environment |
| Isolate 24 | 1 | JF411391 | 99.38 | The single clone with the highest % ID was from soil |
| Isolate 26 | 7 |  | 99.33 | The majority of the clones (6) were from marine environments |
| Isolate 28 | 20 | - | 97.51 | Of the 20 clones, 12 were from groundwater environments, 5 from mining wastewater and 1 from a marine environment |

**Titles to** **Supplementary Figures and Tables**

**Fig. S1** DGGE analysis of enrichments TPW and 2m, demonstrating microbial community structure change under the selective pressure of diamondoid carboxylic acids. Transformation of A1CA over 33 days by TPW (A) and 2m (C) and of 3EA by TPW (B) and 2m (D) communities. Numbers refer to bands identified in Table S2.

**Table S1** Anion and cation analysis of TPW and 2m OSPW samples (ND: Not detected)

**Table S2** Composition of bacterial communities in A1CA and 3EA transforming enrichments with TPW as inoculum (day 0, 11 and 33) based on high-throughput sequencing analysis of 16S rRNA genes. Note that when the same genus is mentioned more than once it indicates different OTUs from the same genus.

**Table S3** Blast*n* analysis of 16S rRNA sequences from DGGE bands excised from the A1CA and 3EA transforming communities derived from samples TPW and 2m

Table S4 BLAST*n* analysis of the 16S rRNA gene sequences obtained from isolates.

Table S5 Source and distribution of environmental sequences with >97% 16S rRNA gene sequence identity to the operational taxonomic units (OTUs) obtained from this study.
